# Tinder Use and Romantic Relationship Formations: A Large-Scale Longitudinal Study

**DOI:** 10.3389/fpsyg.2020.01757

**Published:** 2020-08-14

**Authors:** Eilin K. Erevik, Joakim H. Kristensen, Torbjørn Torsheim, Øystein Vedaa, Ståle Pallesen

**Affiliations:** ^1^Department of Psychosocial Science, University of Bergen, Bergen, Norway; ^2^Department of Health Promotion, Norwegian Institute of Public Health, Bergen, Norway; ^3^Department of Mental Health, Norwegian University of Science and Technology, Trondheim, Norway; ^4^Voss District Psychiatric Hospital, NKS Bjørkeli, Voss, Norway; ^5^Department of Research and Development, St. Olavs University Hospital, Trondheim, Norway; ^6^Optentia, North-West University Vaal Triangle Campus, Vanderbijlpark, South Africa

**Keywords:** romantic relationships, Tinder, students, demographics, personality, mental health, substance use

## Abstract

The current paper aims to investigate if Tinder use predicts romantic relationship formation 1 year later and to identify demographic, personality, mental health, and substance use covariates in the relationship between Tinder use and romantic relationship formation. Data were collected by online surveys (two waves) among students in Bergen, Norway. The first survey was administered during fall 2015 (T1). The follow-up took place 1 year later (fall 2016, T2). The sample consisted of the 5253 participants who reported to be single at T1. The surveys included questions about Tinder use, demographics, personality (the Five-Factor Model’s personality traits), mental health (i.e., symptoms of depression and anxiety), alcohol use, and use of illegal substances. Bivariate comparisons were conducted to assess differences in demographics, personality traits, mental health, and substance use between Tinder users and non-Tinder users. Further, crude and adjusted binary logistic regressions were employed to investigate if Tinder use at T1 predicted romantic relationship formation at T2, when controlling for relevant covariates. Tinder users differed from non-users on almost all included variables. Compared to non-users, Tinder users were younger and more likely to be men, born in Norway, childless, and non-religious. Tinder users had higher scores on extroversion and agreeableness and lower scores on openness compared to non-users. Further, compared to non-users, Tinder users reported more symptoms of anxiety and were more likely to have hazardous, harmful, or dependent alcohol use and to report use of illegal substances. Compared to non-users, Tinder users had a higher likelihood of having formed a romantic relationship at T2 in the crude model (*p* < 0.05) and when controlling for demographic (*p* < 0.05) and mental health (*p* < 0.05) covariates. However, when controlling for personality, substance use, and all included covariates, the difference in likelihood of romantic relationship formation was no longer significant. In conclusion, the current results suggest that Tinder users have a higher likelihood of forming romantic relationship longitudinally, but that this increased likelihood can be explained by Tinder users’ personality and substance use characteristics.

## Introduction

The prevalence rates of singledom and childlessness are increasing in Western and Asian societies (Nargund, 2009; Adamczyk, 2017). Identifying factors that may increase an individual’s likelihood of getting a partner (and, thus, perhaps also children) is of great importance on both an individual and societal level. Over the last years, online dating apps have become an increasingly popular platform for individuals seeking romantic relationships (Smith and Anderson, 2016). One such dating app is Tinder. Tinder is a picture-based dating app for smartphones, with which users are introduced to potential sexual or romantic partners filtered by their preferences in terms of gender, age, and geographical proximity. Potential partners are typically presented with a few photos; a short, written introduction (bio); and information regarding age, gender, and education/work status. When presented with a potential date, users can choose to dismiss them (by swiping left) or to “like” them (by swiping right). If two persons swipe right on each other, they are “matched” and can engage in a conversation per text within the app. The app is mostly used by heterosexual young adults and is currently the most popular dating app with users in more than 190 countries and 1.6 billion “swipes” every day (Duguay, 2017; Timmermans and De Caluwé, 2017; Tinder, 2019).

Being part of a happy, long-term romantic relationship is an important life goal for many people (Reis and Downey, 1999). Most theories on why humans engage in romantic relationship are based on evolutionary psychology, emphasizing romantic relationships as a means of survival (i.e., resource acquisition and protection), reproduction, and care for offspring (Reis and Downey, 1999; Fletcher et al., 2015). The notion of romantic relationships as an evolutionary adaptation is supported by empirical evidence suggesting that people in successful monogamous romantic relationships (and their children) live longer, are happier, and are physically and mentally healthier compared to single/divorced/widowed individuals (Kiecolt-Glaser and Newton, 2001; Fletcher et al., 2015).

The emergence of dating sites and apps, such as Tinder, has changed the dating scene dramatically. With online dating, people are not limited by time, social, and geographical boundaries to the same degree as with off-line dating. Thus, online dating increases the number of potential romantic and sexual partners available (Regan, 2016). Today, more and more individuals regard online dating as a good way to meet potential partners, and the popularity of such apps is increasing (Smith and Anderson, 2016). Location-based, real-time dating apps, such as Tinder, differ from traditional online dating sites by being considered as more casual and effortless to use. In addition, such apps make it more convenient to meet users off-line due to the location filter function that ensures physical proximity (Orosz et al., 2016; Ranzini and Lutz, 2017). The accessibility of Tinder together with the emphasis put on pictures in self-presentations via the app, have fueled the notion that Tinder functions mainly as a an app for seeking casual sex/hookups (Riley, 2015; Sales, 2015; Ranzini and Lutz, 2017; LeFebvre, 2018). Empirical research has, however, shown that the motives for Tinder use are diverse, and motives such as seeking long-lasting relationships and passing time/entertainment greatly surpasses casual sex motives (Hobbs et al., 2017; Sumter et al., 2017; Timmermans and De Caluwé, 2017).

Few studies have investigated whether Tinder facilitates or hampers formation of romantic relationships. One study found a positive association between the number of meetings through Tinder and romantic relationship formation with another Tinder user (Timmermans and Courtois, 2018). Limitations with that study include its cross-sectional design and its lack of a comparison group consisting of non-Tinder users. When exploring possible effects of Tinder use on romantic relationship formation, it is important to control for individual differences in demographics, personality, mental health, and substance use as such characteristics predict romantic relationship formation (Shaver and Brennan, 1992; Buss, 2007; Meyer and Paul, 2011; Petraitis et al., 2014; Erevik et al., 2019). Thus, demographics, personality, mental health, and substance use may act as third variables in the relationship between Tinder use and romantic relationship formation if they predict Tinder use as well.

Few studies have investigated demographic characteristics associated with Tinder use (Sumter and Vandenbosch, 2019). It has been suggested that Tinder use is common among younger adults and men (Timmermans and De Caluwé, 2017; LeFebvre, 2018; Sumter and Vandenbosch, 2019). The proposed overrepresentation of younger adults and men on Tinder has been suggested to be explained by these groups’ interest in casual sex (and Tinder’s reputation as an app via which casual sex can be obtained) (Buss, 1989; Timmermans and De Caluwé, 2017; LeFebvre, 2018; Sumter and Vandenbosch, 2019). In a similar manner, one may expect country of birth and religious identification to predict Tinder use as these characteristics have been found to predict attitudes toward causal sex. Those who are not born in Western countries and religious individuals are typically more skeptical about casual sex and may, thus, be more reluctant to use Tinder (Ahrold and Meston, 2010; Yu, 2010; Adamczyk and Hayes, 2012). Another demographic characteristic that may relate to Tinder use is parental status as one may assume that Tinder may be particularly convenient for single parents due to the limited time they have available for off-line partner searching.

The Five-Factor Model of personality, including extroversion, agreeableness, conscientiousness, neuroticism, and openness, is considered to be the best empirically supported personality taxonomy (McCrae and John, 1992; Larsen et al., 2013). Tinder users have been found to score higher on extroversion and openness and lower on conscientiousness compared to non-users (Timmermans and De Caluwé, 2017). Similar results are found in a study by Carpenter and McEwan (2016) and indicate that users of dating apps (including Tinder) are more sociable and impulsive (i.e., traits related to extroversion), compared to non-users.

To date, there is a dearth of research on dating apps and mental health. No study has specifically explored the associations between Tinder use and depression and anxiety. However, some studies have investigated the associations between online dating and psychological distress with inconsistent findings. Some have found a positive association, and others have found an inverse association (Valkenburg and Peter, 2007; Robinson, 2017). The inconsistencies in previous findings regarding mental health and online dating may reflect differences in samples and types of online dating sites/apps investigated.

Research on dating apps with regard to alcohol and illegal substance use is so far limited to studies investigating the relationship among homosexual men. Robinson (2017) reported in this regard that respondents using both casual sex–seeking sites and dating sites scored higher on alcohol and illegal substance use compared to non-users of such sites. In a study among men using Grindr (a dating/casual sex app for homosexual, bisexual, transgender, and queer people) it was found that 12% reported using the app for finding people with whom to drink and use drugs (Holloway et al., 2014).

Against this backdrop, the current study aimed to investigate if Tinder use predicted formation of romantic relationships (i.e., change in relationship status from single to in a romantic relationship) longitudinally when also controlling for demographic variables (i.e., age, sex, country of birth, parental status, and religious identification), personality traits (i.e., the Five-Factor Model’s personality traits), mental health (i.e., depression and anxiety symptoms), and substance use (i.e., alcohol consumption and use of illegal drugs).

## Materials and Methods

### Procedures and Sample

The present study utilizes data from a larger project investigating health, substance use, and social media use among students (Erevik et al., 2017a). The sample consists of students from the four largest institutions of higher education in Bergen, Norway, including three public institutions—the University of Bergen (UiB), Bergen University College (HiB), and the Norwegian School of Economics (NHH)—and one private institution, Norwegian Business School (BI, campus Bergen). A total of 28,553 students were invited via email to participate in an online survey during fall 2015. A total of 11,236 (39.4% of invited students) participated. The participants who responded to the survey in 2015 (Time 1, T1) were invited to participate in a follow-up online survey (Time 2, T2) during fall 2016. A total of 5217 (51.5%) agreed to participate at T2. Invitations to participate at T2 were sent to the participants’ student emails. However, some participants had ended their education between T1 and T2 (approximately 40% end their education yearly in Bergen), and a large proportion of these did not receive the reinvitation email as their student email account had expired. The T2 response rate is, thus, likely to have been significantly higher than 51.5% among those who received the invitation to participate. The participants were met with an informed consent page on the first page of the surveys, via which they were informed about the study, data storage, and data use procedures and their right to abstain from participation. The project was approved by the Regional Committee for Medical and Health Research Ethics, Western Norway (no. 2015/1154). Those who responded took part in a lottery with two iPhone 6s/7s and 50 gift cards (each with a value of 500 NOK = ∼50 EUR) as prizes. For more information regarding the surveys and sample, see Erevik et al. (2017a).

In the current study, only participants who reported to be single at T1 were initially included, which amounted to 5,253 persons. The sample was further restricted to those who also participated at T2 (*n* = 2,385). These comprised the analytic sample concerning the associations between Tinder use and later romantic relationship formation. The T1 response rate for the current sample is not known as the proportion of single students in the student population is unknown. The T2 response rate for those who reported to be single at T1 was 45.8%.

### Measurements

#### Tinder Use and Demographics

Use of Tinder was measured by asking participants “Which social media sites/apps do you use (you can select several sites)?” (response options: Facebook, Twitter, Instagram, Myspace, Tinder, Snapchat, Jodel, Kik, none, others) (Karl et al., 2010). Demographic variables were measured by closed-ended questions concerning year of birth (response options ranging from 1940 to 2000), sex (response options: woman, man), place of birth (response options: Norway, a Nordic country outside Norway, a European country outside the Nordic countries, Asia, Africa, Central or South America, North America, and Oceania), religious identification (response options: Buddhism, Hinduism, Islam, Judaism, Catholic Christianity, Orthodox Christianity, Protestant Christianity, other, none), and parental status (response options: do not have child/ren, have daily custody of a child/ren, have shared custody of a child/ren, have a child/ren but not custody) at T1 (Nedregård and Olsen, 2014). Participants were asked about relationship status (response options: single, in a relationship but living alone, cohabitant, married/registered partnership, other) at both T1 and T2.

#### Personality

The Five-Factor Model’s personality traits (i.e., extroversion, agreeableness, conscientiousness, neuroticism, and openness) were assessed at T1 using the Mini-International Personality Item Pool (Mini-IPIP) (Donnellan et al., 2006). The Mini-IPIP consists of 20 items (i.e., four items for each trait), and the respondents are asked to rate the degree to which specific statements regarding behavior describes them. Response options range from “very wrong” (1) to “very right” (5). Total scores range between 4 and 20 for each trait, and higher scores indicate higher levels of the personality trait in question. The internal reliability of the measurement was acceptable in the current study. The items measuring extroversion, agreeableness, conscientiousness, neuroticism, and openness had Cronbach’s alphas of 0.83, 0.77, 0.69, 0.75, and 0.74, respectively.

#### Mental Health

Mental health was assessed using the Hopkins Symptoms Check List (HSCL-25) (Derogatis et al., 1974). The HSCL-25 consists of 25 items measuring symptoms of anxiety and depression. When answering the HSCL-25, the participants are asked to indicate to which degree different symptoms of anxiety (e.g., heart palpitations) and depression (e.g., feeling of hopelessness) have bothered them during the past 2 weeks (response options: not at all, a little, quite a bit, extremely). Total scores range between 10 and 40 for anxiety and between 15 and 60 for depression. In the current study, the HSCL-25 obtained Cronbach’s alphas of 0.81 and 0.89 for the subscales measuring symptoms of anxiety and depression, respectively.

#### Substance Use

Alcohol use was assessed by the Alcohol Use Disorders Identification Test (AUDIT), comprising 10 items (Bohn et al., 1995; Babor et al., 2001), Cronbach’s alpha = 0.78 (current study). The test measures three dimensions: consumption (three items: frequency of drinking, quantity consumed, and frequency of heavy drinking), dependency symptoms (three items measuring frequency of impaired control, salience, and morning drinking), and harmful alcohol use (four items measuring frequency of guilt after drinking, blackouts, alcohol-related injuries, and others being concerned about the respondent’s drinking). The response options vary somewhat for the different items with the most common response options being: never, less than monthly, monthly, weekly, daily or almost daily. Total AUDIT scores range from 0 to 40. AUDIT scores of or above 8, 16, or 20 indicate hazardous, harmful, or dependent alcohol use, respectively (Bohn et al., 1995; Babor et al., 2001). Illegal substance use was measured by the question: “Have you ever used drugs?” (yes, no). Those who answered “yes” were further asked: “How many times in the last 6 months have you used the following drugs? (a) hashish/marijuana, (b) ecstasy, (c) LSD/hallucinogens, (d) amphetamine/methamphetamine, (e) ADHD medications (without prescription), (f) cocaine (crack), (g) anabolic steroids, (h) sedatives (without prescription), (i) heroin, and (j) synthetic heroin (without prescription)” (response options: never, I have used before but not in the last 6 months, 1–4 times, 5–50 times, more than 50 times) (Nedregård and Olsen, 2014).

### Analysis

Data analyses were conducted with IBM SPSS Statistics for Mac, Version 25 (IBM Corp., Armonk, NY, United States). Missing data were deleted listwise. Categorical variables were dichotomized before the analyses. The dichotomous variables were sex (man vs. woman), place of birth (countries outside Norway vs. Norway), religious identification (non-religious vs. religious), parental status (do not have children vs. have children), and illegal drug use in the last 6 months (no use vs. use). Relationship status was transformed into a dichotomous variable (0 = single at T1 and T2; 1 = single at T1, relationship at T2) indicating development of a romantic relationship between T1 and T2. In addition, AUDIT scores were transformed into an ordinal variable (0 = no alcohol use, AUDIT = 0; 1 = low-risk alcohol use, 0 < AUDIT < 8; 2 = hazardous alcohol use, 7 < AUDIT < 16; 3 = harmful or dependent alcohol use, AUDIT > 15).

Descriptive analyses were conducted to portray the sample’s central tendencies on the included variables. To check for any potential dropout biases, students who only participated at T1 were compared to students who participated in both waves on the included variables with independent sample *t* and chi-square tests. The effect sizes of significant group differences are reported as Cohen’s *d*s and phi coefficients. By conventional standards Cohen’s *d*s of 0.20, 0.50, and 0.80 represent small, moderate, and large effect sizes, respectively (Cohen, 1988). For phi coefficients, 0.10, 0.30, and 0.50 represent small, moderate, and large effect sizes, respectively (Cohen, 1988).

Further, independent sample *t* and chi-square tests were conducted to compare demographic, personality, mental health, and substance use characteristics between Tinder users and non-Tinder users at baseline (T1), also broken down by sex.

To investigate the association between Tinder use and development of romantic relationship, six binary logistic regression analyses were conducted. The dependent variable was change in relationship status from single at T1 to in a romantic relationship at T2 (coded as “1”), and being single in both waves served as the reference category (coded as “0”). The 19 participants who reported “other” as their civil status at T2 were excluded from the analyses. Crude, partly, and fully adjusted regressions were conducted using baseline scores (T1) as independent variables (IV): In the crude analysis (model 0), only use of Tinder was included as an independent variable. In four partly adjusted models (1–4), Tinder use comprised the independent variable of interest while separately controlling for other groups of IVs. These were demographics (model 1; IV = age, sex, born in Norway, parental status, and religious identification), personality (model 2; IV = extroversion, agreeableness, conscientiousness, neuroticism, and openness), mental health (model 3; IV = depression and anxiety symptoms), and substance use (model 4; IV = no alcohol use, low-risk alcohol use, hazardous alcohol use, harmful or dependent alcohol use, and illegal substance use last 6 months). Finally, a fully adjusted model was run, in which all independent variables were included simultaneously. In addition, we tested for potential interaction effects by sex in all models.

## Results

### Sample and Dropout Analysis

Descriptive analyses revealed that 36.2% (*n* = 1666) of the sample (i.e., the single students) reported using Tinder. The sample’s mean age was 23.3 years (range: 17–75, *SD* = 4.6), 60.0% (*n* = 3150) were women, and the majority were born in Norway (93.4%, *n* = 4090). A total of 2.8% (*n* = 149) reported having children, and 34.1% (*n* = 1,778) reported being religious. Dropout analysis revealed no significant differences between respondents participating only at T1 and respondents participating at both T1 and T2 except the proportion of women and agreeableness scores that were somewhat higher among respondents participating in both waves compared to among those who only participated at T1 (see Table 1).

**TABLE 1:** Sample characteristics and dropout analysis, *N* = 5,253.

	**Full sample**	**Only T1,*n* = 2849**	**T1 and T2,*n* = 2404**	**Significance tests and effect sizes**
	
**Characteristics**	**Mean (*SD*)/%**	**Mean (*SD*)/%**	**Mean (*SD*)/%**	
Tinder use	36.2%	35.1%	37.4%	*NS*
**Demographics**				
Age	23.3 (4.6)	23.3 (4.7)	23.2 (4.5)	*NS*
Women	60.0%	58.3%	62.0%	Phi = 0.038**
Born in Norway	93.4%	92.9%	93.9%	*NS*
Have child/ren	2.8%	3.0%	2.6%	*NS*
Religious	34.1%	34.6%	33.5%	*NS*
**Personality^a^**				
Extroversion	14.0 (3.8)	14.1 (3.8)	13.9 (3.7)	*NS*
Agreeableness	16.6 (2.9)	16.5 (3.0)	16.8 (2.8)	Cohen’s *d* = 0.096**
Conscientiousness	14.3 (3.3)	14.2 (3.3)	14.4 (3.2)	*NS*
Neuroticism	10.8 (3.6)	10.8 (3.6)	10.9 (3.6)	*NS*
Openness	14.6 (3.2)	14.6 (3.2)	14.6 (3.2)	*NS*
**Mental health**				
Depression^b^	24.7 (7.7)	24.7 (7.8)	24.6 (7.7)	*NS*
Anxiety^c^	15.1 (4.1)	15.1 (4.1)	15.1 (4.1)	*NS*
**Substance use**				
No alcohol use	6.2%	6.2%	6.2%	*NS*
Low-risk alcohol use^d^	32.2%	32.4%	31.9%	*NS*
Hazardous alcohol use^*e*^	51.0%	50.0%	52.2%	*NS*
Harmful or dependent alcohol use^*f*^	10.6%	11.4%	9.7%	*NS*
Illegal substance use last 6 months	16.9%	16.9%	16.8%	*NS*

T1, time of the first survey; T2, time of the second survey; SD, standard deviation, **p < 0.01; NS, no significant difference between groups. ^*a*^Total scores range from 4 to 20 for each trait, ^*b*^total scores range from 15 to 60, ^*c*^total scores range from 10 to 40, ^*d*^0 < AUDIT < 8, ^*e*^7 < AUDIT < 16, ^*f*^AUDIT > 15.

### Comparison of Tinder Users and Non-Tinder Users

Comparisons of Tinder users and non-Tinder users are shown in Table 2. There were statistically significant differences between Tinder users and non-Tinder users on most of the included variables. Compared to non-Tinder users, Tinder users were more likely to be younger, men, and born in Norway and less likely to have children and to identify with a religious belief. Tinder users scored higher on extroversion and agreeableness and lower on openness and reported more symptoms of anxiety compared to non-users. Further, Tinder users were less likely to report no or low-risk alcohol use and more likely to report hazardous, harmful, or dependent alcohol use and illegal substance use the last 6 months compared to non-Tinder users. There were no significant differences in scores on conscientiousness, neuroticism, and symptoms of depression between Tinder users and non-Tinder users. The effect sizes of the significant group differences were all small or very small. Similar patterns of differences between Tinder users and non-Tinder users were observed in the analyses that were broken down by sex (results not shown).

**TABLE 2:** Distribution of demographic, personality, mental health, and substance use characteristics among Tinder users (*n* = 1666) and non-Tinder users (*n* = 2940).

	**Tinder users**	**Non-Tinder users**	**Significance tests and effect sizes**
	
**Single respondents**	**Mean (*SD*)/% (95% CI)**	**Mean (*SD*)/% (95% CI)**	
**Demographics**			
Age	22.8 (3.2)	23.5 (5.1)	Cohen’s *d* = 0.148***
Women	56.6%	61.9%	Phi = −0.052**
Born in Norway	95.6%	92.6%	Phi = 0.059***
Have child/ren	1.6%	3.3%	Phi = −0.052**
Religious	28.2%	36.7%	Phi = −0.087***
**Personality^a^**			
Extroversion	14.7 (3.4)	13.5 (3.8)	Cohen’s *d* = 0.320***
Agreeableness	16.7 (2.8)	16.6 (2.9)	Cohen’s *d* = 0.048***
Conscientiousness	14.2 (3.3)	14.4 (3.2)	*NS*
Neuroticism	10.8 (3.6)	10.8 (3.6)	*NS*
Openness	14.4 (3.2)	14.7 (3.2)	Cohen’s *d* = 0.088**
**Mental health**			
Depression^b^	24.8 (7.7)	24.6 (7.6)	*NS*
Anxiety^c^	15.3 (4.1)	15.0 (4.1)	Cohen’s *d* = 0.068*
**Substance use**			
No alcohol use	0.8%	9.1%	Phi = −0.166***
Low-risk alcohol use^d^	22.3%	37.9%	Phi = −0.161***
Hazardous alcohol use^*e*^	62.8%	44.3%	Phi = 0.179***
Harmful or dependent alcohol use^*f*^	14.0%	8.7%	Phi = 0.084***
Illegal substance use last 6 months	21.2%	14.4%	Phi = 0.087***

SD, standard deviation; CI, confidence interval, *p < 0.05, **p < 0.01, ***p < 0.001; NS, no significant difference between groups. ^*a*^Total scores range from 4to 20 for each trait, ^*b*^total scores range from 15 to 60, ^*c*^total scores range from 10 to 40, ^*d*^0 < AUDIT < 8, ^*e*^7 < AUDIT < 16, ^*f*^AUDIT > 15.

### Association Between Tinder Use and Formation of Romantic Relationship

Crude, partly, and fully adjusted regression coefficients of Tinder use on the development of romantic relationships are displayed in Table 3. The crude analysis showed a positive association between Tinder use and romantic relationship formation, indicating increased probability of romantic relationship formation when using Tinder. This association was also significant when adjusting for both demographics and mental health factors (*p* < 0.05). However, when adjusting for personality factors and substance use and when adjusting for all IVs, the association between Tinder use and relationship formation no longer remained significant. None of the sex interaction effects turned out significant (results not shown).

**TABLE 3:** The association between Tinder use and romantic relationship formation.

	**In a romantic relationship at T2, reference category: single at both T1 and T2 (OR = 1.00)**	**Models**
	**OR (95% CI)**	
**Crude analysis**		
Tinder use	1.31 (1.06–1.62)*	χ^2^ = 6.10, *df* = 1
		Cox and Snell = 0.003;
		Nagelkerke *R*^2^ = 0.004
**Adjusted for demographics^1^**		
Tinder use	1.30 (1.04–1.61)*	χ^2^ = 12.41, *df* = 6
		Cox and Snell = 0.006;
		Nagelkerke *R*^2^ = 0.009
**Adjusted for personality^2^**		
Tinder use	1.18 (0.95–1.47)	χ^2^ = 44.33, *df* = 6
		Cox and Snell = 0.020;
		Nagelkerke *R*^2^ = 0.032
**Adjusted for mental health^3^**		
Tinder use	1.31 (1.06–1.62)*	χ^2^ = 6.99, *df* = 3
		Cox and Snell = 0.003;
		Nagelkerke *R*^2^ = 0.005
**Adjusted for substance use^4^**		
Tinder use	1.13 (0.90–1.42)	χ^2^ = 34.06, *df* = 5
		Cox and Snell = 0.016;
		Nagelkerke *R*^2^ = 0.025
**Fully adjusted^5^**		
Tinder use	1.08 (0.85–1.35)	χ^2^ = 64.37, *df* = 17
		Cox and Snell = 0.030;
		Nagelkerke *R*^2^ = 0.047

Binary logistic regression analyses, n = 2385. T1, time of the first survey; T2, time of the second survey; OR, odds ratio; CI, confidence interval, *p < 0.05. ^1^Adjusted for age, sex, country of birth, parental status, and religiousness; ^2^adjusted for extroversion, agreeableness, conscientiousness, neuroticism, and openness; ^3^adjusted for symptoms of depression and anxiety; ^4^adjusted for alcohol and illegal substance use; ^5^adjusted for demographics, personality, mental health, and alcohol/illegal substance use.

## Discussion

The current study investigates associations between individual characteristics and Tinder use. Many of these characteristics have not been investigated in relation to Tinder use previously, and the current study contributes such with several novel results. Further, the current study is the first to investigate the relationship between Tinder use and romantic relationship formation while including a control group of non-Tinder users. Differences between Tinder users and non-Tinder users are addressed first in the following discussion before the associations between Tinder use and romantic relationship formations are considered.

### Differences Between Tinder Users and Non-Tinder Users

The results concerning differences in demographics between Tinder users and non-users mirrors previous research, lending support to the notion that Tinder users are in general younger compared to non-users (Smith and Anderson, 2016). In addition, users were more likely to be men, born in Norway, non-religious, and not to have children. The association between Tinder use and male sex, being born in Norway, and non-religiosity could relate to Tinder’s reputation as a hookup app because male, Norwegian-born, and non-religious students have been shown to be more positive toward casual sex compared to their counterparts (Ahrold and Meston, 2010; Yu, 2010; Adamczyk and Hayes, 2012; LeFebvre, 2018). The finding that students with children were less likely to use Tinder compared to students who did not have children can be speculated to reflect that students with children may have less time available for dating, both off-line as well as online.

The results concerning the associations between personality traits and Tinder use support the findings by Timmermans and De Caluwé (2017), who found Tinder users to have higher scores on extroversion compared to non-users. The aforementioned study found no significant association between Tinder use and agreeableness and a positive association between Tinder use and scores on openness. In contrast, Tinder users in the current study scored higher on agreeableness and lower on openness compared to non-users. The explanations as to why agreeable students were more likely to use Tinder are not apparent. The positive association between agreeableness and Tinder use is also somewhat at odds with the results of a recent study that found Tinder users to have higher scores on the Dark Triad traits (i.e., Machiavellianism, narcissism, and psychopathy, which are all inversely associated with agreeableness) (Jakobwitz and Egan, 2006; Sevi, 2019). The differences between the findings of the current study and those of Sevi (2019) may be explained by differences in the samples. The current study’s sample consisted of relatively young and single individuals, and the sample in Sevi’s (2019) study consisted of somewhat older adults (mean age was 30.78) including both single and non-single individuals. It is reasonable to expect that Tinder users who are in romantic relationships may have lower agreeableness scores as they are likely to look for an affair. One possible explanation for the positive association between agreeableness and Tinder use observed in the current study could relate to agreeable individuals’ tendency to attribute good intentions to others (McCrae and John, 1992). Agreeable individuals’ trust in others may involve them being less concerned with being deceived on Tinder and, thus, more positive toward the app. Individuals with higher scores on openness are characterized by an interest in novel experiences (McCrae and John, 1992). Given that 36.2% of the sample used Tinder, Tinder use may be viewed as rather conventional by the students; hence, lack of novelty may explain the observed inverse relationship between Tinder use and openness.

In the current study, Tinder users reported more symptoms of anxiety compared to non-users. To our knowledge, no previous study has investigated the association between anxiety and Tinder use, specifically. The current findings are in line with findings from a study investigating levels of depression and anxiety among gay, bisexual, and queer men using dating or casual sex–seeking sites (Robinson, 2017). One possible explanation for the association between anxiety and Tinder use may be that individuals with more anxiety are more likely to be online daters because such dating may involve less evaluation apprehension compared to face-to-face dating. This social compensation hypothesis is, however, not supported by empirical findings (Valkenburg and Peter, 2007). Another possible explanation for the observed association between anxiety and Tinder use is that characteristics of Tinder specifically, in particular its emphasis on photos, may increase anxiety through intensifying negative perceptions toward one’s own appearance (Strubel and Petrie, 2017).

In the current study, Tinder users had higher alcohol consumption and were more likely to have used illegal substances the last 6 months compared to non-users, both of which are novel findings. Still, similar results have been reported in studies on dating sites/apps for homosexual, bisexual, transgender, and queer people (Holloway et al., 2014; Robinson, 2017). The associations between alcohol and substance use and Tinder use have several possible explanations. For one, individuals who have a higher consumption of alcohol and illegal substances may be more likely to use Tinder as they may use the app to find others with whom to drink or take illegal substances (Holloway et al., 2014). Another possible explanation could be that using Tinder leads to an increase in alcohol and illegal substance use as alcohol and other substances could be used to reduce anxiety in relation to dating (Monahan and Lannutti, 2000; Isaiah Green, 2003). It is also reasonable to expect that most dates take place on alcohol-serving premises. Finally, it is also possible that Tinder users have some characteristics that may make them more likely to both use Tinder and have a higher consumption of alcohol and drugs (e.g., extroversion and sensation seeking) (Erevik et al., 2017a, b; Timmermans and De Caluwé, 2017).

### Tinder Use and Formation of Romantic Relationships

Tinder users were more likely to have formed a romantic relationship 1 year later compared to non-users when no covariates were controlled for. This association was upheld when demographical and mental health characteristics were controlled for, suggesting that Tinder users’ increased likelihood of forming romantic relationships could not be explained by their demographical or mental health characteristics. However, there were no significant differences between Tinder users and non-users in terms of likelihood of forming romantic relationships when personality characteristics or substance use characteristics were controlled for or in the fully adjusted analysis. These findings suggest that the observed increased likelihood of forming romantic relationships found among Tinder users may be explained by their personality and substance use characteristics.

The finding that Tinder use was not associated with romantic relationship formation when personality traits were controlled for suggests that personality characteristics associated with Tinder use may explain the observed (crude) association between Tinder use and romantic relationship formation. In the current study, Tinder users had higher scores on extroversion and agreeableness and lower scores on openness compared to non-users. Extroversion has consistently been found to be perceived as an attractive trait in a partner, and extroverts have a higher likelihood of entering romantic relationships (Botwin et al., 1997; Figueredo et al., 2006; Neyer and Lehnart, 2007; Erevik et al., 2019). Higher agreeableness scores (although typically perceived as attractive) and lower openness scores have, however, been associated with a lower likelihood of forming romantic relationships (Botwin et al., 1997; Figueredo et al., 2006; Stavrova and Ehlebracht, 2015; Erevik et al., 2019). Based on previous findings, it, thus, seems plausible to assume that it is the Tinder users’ extroversion scores that explain why the association between Tinder use and romantic relationship formation was non-significant when personality traits were controlled for. An *ad hoc* test was conducted including the personality traits separately as covariates. The association between Tinder use and romantic relationship formation remained significant when agreeableness, conscientiousness, neuroticism, and openness were controlled for but not when extroversion was controlled for (results not shown). Thus, the *ad hoc* testing suggests that it is the differences in extroversion scores between Tinder users and non-users that explain the dating success of the former group.

Tinder use was not associated with romantic relationship formation when alcohol and substance use were controlled for; hence, alcohol and substance use characteristics associated with Tinder use may explain the (crude) association between Tinder use and romantic relationship formation. In the current study, Tinder users had higher consumption of alcohol and illegal substances compared to non-Tinder users. This suggests that Tinder users’ heightened substance use might be the reason for their relationship success. This claim is supported by the findings of a previous study based on the same sample as the current one, which found alcohol and illegal substance use to be a positive predictor of romantic relationship formation (Erevik et al., 2019). It should be noted, however, that a positive association between alcohol and substance use and romantic relationship formation has not been a consistent finding in the literature (Kandel et al., 1986; Fu and Goldman, 1996; Leonard and Rothbard, 1999). Thus, one could assume that alcohol and illegal substance use may only predict romantic relationship formation in certain types of populations, such as student populations in which alcohol use, including heavy alcohol use, is the norm and in which severe and detrimental alcohol and drug problems might be less common (Erevik et al., 2017a).

### Implications

The current findings have implications for future research. The observed associations between Tinder use and individual characteristics in terms of demographics, personality, mental health and alcohol and substance use, suggest that future studies investigating outcomes related to Tinder use, should include and control for these variables. The current findings also raise some research questions for future studies. An investigation of the cross-lagged relationship between alcohol use and mental health and Tinder use could better elucidate the temporal relationship between the variables in question. Future studies may also investigate possible mediation pathways between Tinder use, personality, substance use, and romantic relationship formation, e.g., if substance use mediates the relationship between Tinder use and romantic relationship formation. Further, even if Tinder use did not predict romantic relationship formation in the current study when covariates were controlled for, it is still possible that certain subgroups may have an increased likelihood of romantic relationship formation via Tinder as compared to other arenas for dating (Grøntvedt et al., 2020). Investigating the associations between Tinder use and romantic relationship formations in such subgroups could be an interesting inquiry for future studies. Subgroups that may have a higher likelihood of romantic relationship formation through Tinder, compared to other arenas, include individuals who have a hard time approaching potential romantic partners in off-line settings or who seldomly seek settings in which one could meet a partner, e.g., introverts or alcohol abstainers.

### Limitations and Strengths

Both some limitations and strengths of the presented study should be noted. The survey was not created to investigate Tinder use specifically; hence, regrettably, we do not have data measuring the participants’ motives for Tinder use (Timmermans and De Caluwé, 2017). Exploring this in future studies would offer an opportunity to investigate whether different motives for Tinder use are associated with different rates of success in terms of romantic relationship formation. Further, an important limitation is that we do not know if those who formed romantic relationship met their partner trough Tinder or if their partner even used Tinder before they met. Another limitation is lack of information regarding details about the nature of the romantic relationships (e.g., if it was a short- or long-term relationship and the degree of romantic love/passion the participants felt toward their partner). The participants who had formed romantic relationships were, however, likely to have had intentions of entering a long-term, romantic relationship as Norwegians usually understand the phrase “being in a relationship” as referring to a serious, monogamous, interpersonal, long-term commitment. There are also potential issues concerning the generalizability of the findings as the sample consisted only of relatively young students in Norway. Tinder is, however, mainly used by young individuals, which suggest that the finding may be relevant for most Tinder users (Smith and Anderson, 2016). Despite these limitations, a major asset of the current study is the relatively large sample size and its longitudinal design. Further, the research questions were novel; hence, the current study contributes such with several unique findings (e.g., the associations between mental health, alcohol and substance use, and Tinder use).

## Conclusion

The current results suggest that Tinder users have a higher likelihood of forming romantic relationship but that this may be explained by Tinder users’ personality, in particular, their higher extroversion scores as well as their substance use characteristics. The results also suggest that Tinder users differ from non-users in terms of demographical, personality, mental health, and alcohol and substance use characteristics.

## Data Availability Statement

The data analyzed in this study is subject to ethical restrictions. Requests to access these datasets should be directed to EE, eilin.erevik@uib.no.

## Ethics Statement

The studies involving human participants were reviewed and approved by Regional Committee for Medical and Health Research Ethics, Western Norway. The patients/participants provided their written informed consent to participate in this study.

## Author Contributions

SP, EE, and TT conceived, designed, and preformed the research. EE and JK conceived and designed the specific study and analyzed the data. JK, EE, SP, ØV, and TT contributed to the writing of the manuscript. All authors contributed to the article and approved the submitted version.

## Conflict of Interest

The authors declare that the research was conducted in the absence of any commercial or financial relationships that could be construed as a potential conflict of interest.
